# Identification of chronic kidney disease risk in relatively lean Southern Chinese: the hypertriglyceridemic waist phenotype vs. anthropometric indexes

**DOI:** 10.1007/s40519-017-0476-8

**Published:** 2018-01-25

**Authors:** Chaomin Zhou, Yongqiang Li, Xiaofei Shao, Hequn Zou

**Affiliations:** 1 Department of Nephrology, The People’s Hospital of Guizhou Province, Guiyang, 550002 China; 2 Department of Nephrology, Institute of Nephrology and Urology, The Third Affiliated Hospital of Southern Medical University, Guangzhou, 510630 China

**Keywords:** Chronic kidney disease, Visceral adiposity, Hypertriglyceridemic waist phenotype, Anthropometric indexes

## Abstract

**Purpose:**

Assessing and comparing the ability of the hypertriglyceridemic waist (HW) phenotype and anthropometric obesity indexes to identify subjects at high risk of chronic kidney disease (CKD) in a relatively lean population in South China.

**Methods:**

Using data from a community-based, cross-sectional study conducted in Zhuhai City, Southern China, we examined associations between the HW phenotype, anthropometric obesity indexes, and incident CKD risk in a relatively lean population. Multiple logistic regression analyses were used to evaluate the associations.

**Results:**

The HW phenotype associated with CKD significantly in the unadjusted analysis (OR 3.53, 95% CI 1.65–7.52, *P* = 0.001). Further adjustment for gender, age, and other potential confounding variables had an impact on the odd ratios (OR); the OR decreased but still existed (OR 2.91, 95% 1.23–6.87, *P* = 0.016). The association of the HW phenotype with CKD remained significant after further adjustment for hypertension and diabetes. No significant association between the anthropometric indexes and incident CKD was found.

**Conclusion:**

The HW phenotype, but not the anthropometric indexes, is associated with an elevated risk of CKD in relatively lean subjects. The HW phenotype appears to be a better predictor of CKD than the anthropometric indexes.

**Level of evidence:**

Level V, descriptive study.

## Introduction

It is widely known that obese subjects are at increased health risk for many diseases such as cardiovascular disease, diabetes, and chronic kidney disease (CKD) [1–3]. Recent meta-analysis even suggests that obesity predicts onset of CKD in the general population [4]. A recent study suggests that body mass index (BMI) is an independent predictive factor for kidney function and BMI was negatively associated with estimated glomerular filtration rate (eGFR). BMI is a widely accepted measurement for assessing overweight and obesity. However, published data suggest that it is waist circumference (WC), but not the commonly used BMI is the best measure to explain obesity-related health risk [5]. Published data suggest that central obesity is more pathogenic than overall obesity. BMI is widely used to evaluate overall obesity, and anthropometric obesity indexes including WC, waist-to-hip ratio (WHR), and waist-to-height ratio (WHtR) are recognised as reliable measures of abdominal adiposity/central obesity. A large number of subjects in China with normal BMI are suffering from abdominal/central obesity and the trends are rising [6]. In addition, in recent years, increasingly published data indicate that despite having normal BMI, the metabolically obese normal-weight (MONW) individuals exhibited a cluster of cardiovascular risk factors and increased incidences of diabetes, cardiovascular diseases, and all-cause mortality [7–9]. Obesity-related health risk may thus be underestimated in these subjects with relatively low BMI.

Though WC is a better predictor of obesity-related disease including CKD compared to BMI, it cannot discriminate between visceral and subcutaneous fat depots. Several studies have suggested that visceral fat is more strongly associated with metabolic risk factors than subcutaneous abdominal fat and visceral fat accumulation directly involved in the pathogenesis of insulin resistance and associated metabolic diseases [10–13]. Increased estimated visceral adipose tissue is associated with decreased eGFR in the early stages of CKD [14]. Asian population including Chinese appear to be more prone to have visceral fat accumulation despite having generally low BMI [15].The hypertriglyceridemic waist (HW) phenotype is thought to be a simple but sensitive surrogate marker of visceral adiposity [16], and our previous study showed that the HW phenotype was associated with CKD in the population aged 40 years and older [17]. It is still not known, however, whether the HW phenotype can predict future CKD in relatively lean (lean subjects or subjects with normal BMI) populations.

We evaluated and compared the ability to identify CKD risk between HW phenotype and classical anthropometric obesity indices in relatively lean Southern Chinese adults.

## Methods

### Study population

Data used in the current study were drawn from a population based, cross-sectional survey conducted in Wanzhai Town, Zhuhai City, a coastal city in South China. The survey was conducted between June 2012 and October 2012. Detailed descriptions of the survey have been reported in our previous paper [18, 19]. In short, participants were selected with a multi-stage stratified random cluster sampling method. Step 1, two communities were selected randomly from Wanzhai Town; step 2, in each of the two selected communities, 500 families were randomly sampled as the target family; and step 3, all the residents in the selected families were sampled. Participants were only permitted to participate in the current analysis if they had a BMI < 24 kg/m^2^.

The Ethics Committee of The Third Affiliated Hospital of Southern Medical University, Guangzhou, approved this study. This study was performed fulfilling the principles of Helsinki Declaration and the International Guidelines for Ethical Review for Epidemiological Studies. All participants gave their informed consent.

### Data collection

A detailed questionnaire was administered by a trained interviewer including physicians, medical students, and nurses before clinical measurements and blood collection. The questionnaire recorded information on demographic data, personal history (coronary artery disease, hypertension, and diabetes), family history in first degree relatives and details about lifestyle (current or past cigarette smoking, alcohol consumption, diet habits, educational status, and physical activity), and so on.

### Physical measurements

Physical measurements, such as body weight, height, waist circumference, and blood pressure (BP), were conducted in the community clinics in the morning between 08:00 a.m. and 11:00 a.m. by physicians, medical students, and nurses, who had received intensive training. Body height and weight were measured with subjects minimally clothed without shoes, using a calibrated clinical scale and stadiometer. Circumference and height were measured to the nearest 0.1 cm and weight to the nearest 0.1 kg. WC was taken midway between the last rib and iliac crest with the participants standing with light garments and breathing out gently. Hip circumference was measured at the widest point around the buttocks. All measurements were taken twice. The average of the two closest measurements was used for analysis. A tolerance limit of 1 cm was set for height and circumference measurements and 1 kg for weight measurement. A third measurement was needed if the difference of the first two measurements was greater than the tolerance limit. BMI was calculated as weight (kg) divided by height squared (m^2^). WHR was calculated as WC (cm) divided by hip circumference (cm) and WHtR was calculated as WC divided by height (cm). BP was measured three times, on the right arm, using a standardized mercury sphygmomanometer after a 15-min rest in the sitting position. The average of the three values was used as the BP for the participant.

### Laboratory assays

Blood specimens were drawn between 7:00 and 9:00 a.m. from all study participants after at least 10 h overnight fasting. First, morning urine samples were collected from all participants, except those women who were actively menstruating or individuals having urinary tract infection symptoms. All the blood and urine analyses were undertaken at the central laboratory in the Third Affiliated Hospital of Southern Medical University. All specimens were transported within 3 h from collection sites and stored at 4 °C until analysis. Serum high-density lipoprotein (HDL) was determined enzymatically with commercially available reagents (Shanghai Gensource Co., Ltd, Shanghai, China), and cholesterol and triglyceride (TG) levels were determined enzymatically with commercially available reagents (Roche Diagnostics, Mannheim, Germany). Blood glucose level was measured with a hexokinase enzyme reference method and serum creatinine (Scr) with an enzymatic method on an autoanalyzer (Hitachi 7170, Hitachi, Tokyo, Japan) [18]. Enzymatic turbidimetric immunoassay method was used to analyse serum levels of high sensitivity C-reactive protein. Urinary albumin and creatinine were measured from a fresh morning spot urine sample or first morning urine sample stored at + 4 °C for less than 1 week. Albuminuria was determined with immunoturbidimetric tests (Audit Diagnostics, Cork, Ireland). Urinary creatinine was measured with Jaffe’s kinetic method [20]. The urinary albumin-to-creatinine ratio (ACR; mg/g creatinine) was calculated.

### Determination of hypertension and diabetes

Participants with a blood pressure (BP) of 140/90 mmHg or higher and/or self-reported diagnosis of hypertension would be considered as having hypertension. Diabetes mellitus was defined as a fasting serum glucose ≥ 7.0 mmol/L and/or self-reported diagnosis of diabetes.

### Definition of the HW phenotype

The HW phenotype was defined as elevated waist circumference (90 cm in men and 85 cm in women), along with an elevated plasma triglyceride (TG) concentration (2.0 mmol/L) [17].

### Determination of CKD

A formula from the Chinese-Modification of Diet Renal Disease (C-MDRD) study: GFR (mL/min/1.73 m^2^) = 175 × (Scr)^−1.234^ × (Age)^−0.179^ × (if female, × 0.79) was used to calculate the estimated glomerular filtration rate (eGFR) [21]. CKD was defined as eGFR less than 60 mL/min/1.73 m^2^ and/or urinary albumin-to-creatinine ratio (ACR) higher than 30 mg/g.

### Statistical analysis

Statistical analyses were conducted with SPSS software (version 13.0). Participants were divided into three groups according to their gender-specific waist circumferences and TG levels. Group 1, waist circumference < 90 cm in men or < 85 cm in women along with TG < 2 mmol/L for both genders; Group 2, solely WC increase (waist circumference ≥ 90 cm in men or ≥ 85 cm in women, along with triglycerides < 2 mmol/L) or solely TG increase (waist circumference < 90 cm in men or < 85 cm in women along with triglycerides ≥ 2 mmol/L); Group 3, increased WC and TG level (waist circumference ≥ 90 cm in men or ≥ 85 cm in women and triglycerides ≥ 2 mmol/L) [22, 23]. Basic characteristics of the participants were listed in Table 1. Continuous variables were presented as medians (25th–75th percentiles) if they had skewed distribution. Mean ± standard deviation would be presented if the continuous variables had a normal distribution. Categorical variables were expressed as percentages. Differences in the three groups were assessed using the Chi-squared test for categorical variables and one-way ANOVA or Wilcoxon rank-sum test for continuous variables.

**Table 1:** Characteristics of subjects in different groups stratified by the hypertriglyceridemic waist phenotype

	Group 1, *n* = 740	Group 2, *n* = 328	Group 3, *n* = 104	*P*
Age (years)	49.05 ± 15.96	55.15 ± 14.07	60.32 ± 13.11	< 0.001
Male (%), *N*	(36.8) 272	(29.9) 98	(20.2) 21	< 0.001
Clinical characteristics				
Hypertension history (%), *N*	(11.2) 82	(16.8) 55	(26.9) 28	< 0.001
Diabetes history (%), *N*	(3.3) 24	(8.3) 27	(8.7) 9	< 0.001
Coronary heart disease history (%), *N*	(2.0) 15	(2.1) 7	(1.9) 2	0.98
Current smoker (%), *N*	(12.7) 93	(9.8)32	(9.8)10	0.34
Current alcohol use (%), *N*	(21.5) 158	(21.1)69	(20.6)21	0.9
Educational status (≥ high school) (%), *N*	(46.3) 328	(36.1) 113	(29.9) 29	< 0.001
Systolic blood pressure (mmHg)	120.81 ± 20.71	129.09 ± 19.03	133.75 ± 19.11	< 0.001
Diastolic blood pressure (mm Hg)	74.19 ± 11.33	76.92 ± 10.56	78.77 ± 12.68	< 0.001
Body mass index (kg/m^2^)	20.93 (19.38–22.37)	22.52 (21.08–23.31)	22.72 (21.88–23.44)	< 0.001
Waist circumference(cm)	76 (71–80)	85 (81-88.75)	91 (87–94)	< 0.001
Hip circumference(cm)	88.92 ± 5.99	92.14 ± 6.07	92.98 ± 7.53	< 0.001
Laboratory values				
Fasting glucose (mmo/L)	4.61 (4.31–4.9)	4.86 (4.5–5.32)	4.93 (4.57–5.58)	< 0.001
Serum C-reactive protein (mg/L)	0.6 (0.31–1.31)	1.02 (0.57–2.16)	1.56 (0.96–2.96)	< 0.001
Serum triglyceride (mmol/L)	0.97 (0.74–1.24)	2.06 (1.23–2.60)	2.44 (2.28–3.01)	< 0.001
Serum low-density lipoprotein (mmol/L)	2.94 (2.46–3.52)	3.24 (2.61–3.88)	2.97 (2.48–3.55)	< 0.001
Serum high-density lipoprotein (mmol/L)	1.64 ± 0.33	1.53 ± 0.32	1.46 ± 0.38	< 0.001
Very low-density lipoprotein (mmol/L)	0.44 (0.34–0.56)	0.92 (0.55–1.16)	1.11 (1.02–1.33)	< 0.001
Serum creatinine (umol/L)	70.82 ± 15.57	74.02 ± 18.83	74.65 ± 14.62	0.012
eGFR (mL/min/1.73 m^2^)	104.04 ± 22.3	100.17 ± 22.37	96.55 ± 21.68	0.001
ACR (mg/g)	7 (5–12)	8 (5-16.5)	8 (6–30)	0.003
Serum uric acid (umol/L)	321.85 ± 84.9	344.37 ± 89.61	372.87 ± 99.67	< 0.001
CKD (%)	(8.7) 61	(12.8) 40	(18.3) 17	0.007

Date expressed as mean ± standard deviation, or number (percentage). Group 1, WC < 90 cm in men or < 85 cm in women along with TG < 2 mmol/L for both genders; group 2, solely WC increase or solely TG increase; group 3, elevated TG levels and elevated WC. Chi-squared test was used for categorical variables and one-way ANOVA or Wilcoxon rank-sum test for continuous variables. Statistically significant (*P* < 0.05)

*ACR* urinary albumin-to-creatinine ratio, *CKD* chronic kidney disease, *eGFR* estimated glomerular filtration rate

Multiple logistic regression models were used to examine the association between the HW phenotype and CKD. Subjects with both low WC and TG, i.e., group 1 was considered as the reference group. A two-tailed *P* value < 0.05 was considered to be significant.

## Results

There were 2142 study subjects in our initial study. All participants were Han ethnic. 308 subjects were excluded from the current study due to missing data on serum fasting glucose, serum creatinine, TG, etc. Participants who received a diagnosis of overweight or obese were also excluded from the current study (*n* = 662). According to the criteria recommended by the Working Group on Obesity in China [24], individuals with baseline BMI > or = 24 kg/m^2^ were considered overweight or obese. At last, 1172 relatively lean adult subjects with baseline BMI < 24.0 kg/m^2^ were enrolled in our study. Among these relatively lean subjects, 740 participants who had low WC and TG levels were assigned to Group 1, 328 participants were assigned to Group 2 and 104 participants met the HW phenotype criteria and were assigned to Group 3. Only 391 men were included in our study.

### Baseline characteristics of the participants

Baseline characteristics of the participants in different groups based on the HW phenotype were shown in Table 1. Subjects with the HW phenotype had a higher mean age, higher systolic and diastolic blood pressure levels, greater WC and hip circumference, and BMI (*P* < 0.001). There is no difference in current alcohol use (%), current smoker (%), and coronary heart disease history (%) among the three groups. Subjects with the HW phenotype had a lower educational level and were more likely to have a history of hypertension. Levels of serum uric acid, triglyceride, C-reactive protein, fasting glucose, and very low-density lipoprotein were significantly higher in subjects with the HW phenotype than those without this phenotype (*P* < 0.001), while serum levels of high-density lipoprotein in these participants were significantly lower (*P* < 0.001).

### Prevalence of CKD based on the HW phenotype

As shown in Table 1, there were still quite a few participants suffering from CKD, though they were not obese according to the criteria recommended by the Working Group on Obesity in China [24]. The prevalence of CKD was 18.3% in subjects with the HW phenotype (Group 3), which was significantly higher than those without this phenotype (*P* < 0.001). The prevalence of CKD in Group1 and Group 2 was 8.7 and 12.8%, respectively.

### Associations of the HW phenotype with CKD

Multivariate-adjusted odds ratios [and 95% confidence intervals (CI)] for CKD according to different phenotypes of serum TG levels and WC are summarized in Table 2. The HW phenotype associated with CKD significantly in the unadjusted model (OR 3.53, 95% CI 1.65–7.52, *P* = 0.001). After adjusting for gender, age, history of stroke, BMI, and other potential confounders, the OR of CKD associated with the HW phenotype was 2.91 (95% CI 1.23–6.87). The association persisted, although it was attenuated after additional adjustment for potential intermediate variables, including hypertension and diabetes.

**Table 2:** Association between the hypertriglyceridemic waist phenotype and CKD in relatively lean subjects

	Model one^a^		Model two^b^		Model three^c^		Model four^d^	
	OR (95% CI)	*P* value	OR (95% CI)	*P* value	OR (95% CI)	*P* value	OR (95% CI)	*P* value
Group 1 (*n* = 740)	Reference		Reference		Reference		Reference	
Group 2 (*n* = 328)	1.78 (1.15–2.76)	0.01	1.45 (0.93–2.28)	0.1	1.67 (1.02–2.75)	0.04	1.52 (0.91–2.53)	0.11
Group 3 (*n* = 104)	3.53 (1.65–7.52)	0.001	2.52 (1.15–5.5)	0.02	2.91 (1.23–6.87)	0.016	2.73 (1.13–6.62)	0.025

^a^Unadjusted

^b^Adjusted for sex, age

^c^History of stroke, history of coronary heart disease, current smoker, current alcohol use, physical inactivity, education attainment, C-reactive protein, serum uric acid, and body mass index

^d^Adjusted for above + diabetes and hypertension

### Associations of anthropometric obesity indexes with CKD

Anthropometric obesity indexes such as WC, WHR, and WHtR were widely considered as surrogate makers of central obesity. We further analysed the relationship between these indexes and CKD in relatively lean Southern Chinese.

As shown in Table 3, there was no statistically significant association between the anthropometric indexes and CKD (*P* > 0.05). Models could not be established even in the unadjusted analysis.

**Table 3:** Logistic regression analysis of the associations between anthropometric indexes and CKD in relatively lean subjects

	Waist circumference		Waist-to-hip ratio		Waist-to-height ratio	
	OR (95% CI)	*P* value	OR (95% CI)	*P* value	OR (95% CI)	*P* value
All participants	1.001 (0.996–1.005)	0.75	1.05 (0.68–1.64)	0.82	1.16 (0.57–2.34)	0.69
Male	0.999 (0.99–1.008)	0.85	0.95 (0.43–2.08)	0.89	0.92 (0.24–3.52)	0.91
Female	1.002 (0.996–1.008)	0.61	1.11 (0.62–2.01)	0.72	1.36 (0.57–3.27)	0.49

## Discussion

To the best of our knowledge, this is the first study that applying the HW phenotype, a surrogate marker of visceral obesity, for assessment of CKD risk in relatively lean subjects. We found that the HW phenotype was independently associated with an increased risk for incident CKD even in non-obese Chinese adults. Subjects with the HW phenotype had a nearly 3.5-fold greater risk of developing CKD than those without this phenotype despite having relatively low BMI. We also examined the associations between the anthropometric obesity indexes (WC, WHR, and WHtR) and CKD risk in the relatively lean population. We found only weak, non-significant associations between these indexes and incident CKD.

It is widely known that obesity is associated with an increased risk of chronic diseases including CKD [4]. With the rapid economic growth and an accelerated pace of nutrition transition, the increasing prevalence of obesity in China is especially alarming. BMI serves as the sole indicator for the standardized definition of obesity [24]. It is generally recommended to consider Chinese as normal if their BMI is > 18.5 and < 24.0 kg m^−2^. Many studies, however, have suggested that BMI is just a measure of general adiposity/overall obesity and central obesity is more pathogenic than overall obesity [25–27]. Recent data have suggested that central body fat distribution, independent of BMI, is associated with an unfavourable pattern of renal hemodynamic [28]. This result is consistent with a previous study, which suggests that a central body fat distribution is related to renal function impairment, even in lean subjects [29]. Studies also suggest that larger increase in central obesity may not be related to the change in BMI [30]. It is thus very likely that a large number of subjects with normal BMI may have central obesity, and this is confirmed by Tingting Du, etc [6].

WC, WHtR, and WHR, surrogate markers of central adiposity, are reported to be associated with incident CKD in the general population [1, 31]. However, a few studies have assessed the relationships between the anthropometric indexes and CKD in a relatively lean population. In our present study, no significant associations between these indexes and incident CKD were found. Our results suggest that the anthropometric central obesity indicators (WC, WHR, and WHtR) failed to have the ability to predict the presence of CKD in the relatively lean population, although they are reported to be associated with incident CKD in the general population.

WC is widely accepted as the best measure of abdominal adiposity [32]. It is a better indicator that explains obesity-related health risk, compared with BMI [5]. However, it fails to distinguish subcutaneous adiposity from visceral adiposity. The key role of visceral obesity has been emphasized recent years. Several studies suggest that it is visceral obesity, but not subcutaneous adiposity correlates with metabolic abnormalities [33, 34]. Surgical removal of visceral fat could lead to metabolic improvements, while removal of abdominal subcutaneous fat in obese subjects could not result in the same improvements [35, 36]. Published data also suggest that visceral fat is more strongly associated with cardiovascular disease than subcutaneous abdominal fat [11]. Imaging techniques, such as computed tomography (CT), magnetic resonance imaging (MRI), and ultrasonography [37], can distinguish visceral from subcutaneous adipose tissue precisely, but they are time-consuming, relatively expensive, and expose the patient to radiation. Reliable surrogate markers of visceral adiposity may thus be particularly useful.

The HW phenotype has been confirmed to be a reliable marker of visceral obesity. Several studies have suggested that the HW phenotype is significantly associated with CVD risk and early diabetic nephropathy [38–41]. Our previous study also suggests that the HW phenotype is associated with CKD in the population aged 40 years and older [19]. Whether the HW phenotype can predict future CKD in relatively lean populations is not known. Chinese are more likely to have visceral fat accumulation despite having generally low BMI [15]. 8.9% (104) subjects in our study showed the HW phenotype. In another word, many subjects in our study were viscerally obese despite having relatively low BMI and this supported the results of Lear, etc [15]. Participants with the HW phenotype had significantly higher prevalence of all metabolic risk factors (larger WC, higher blood pressure, higher levels of serum uric acid, TG, C-reactive protein, fasting glucose, very low-density lipoprotein, and lower high-density lipoprotein) than did those without the HW phenotype. This was consistent with a previous study [42]. Logistic regression analysis suggested that the HW phenotype associated with an increased risk for CKD even in a relatively lean population. The relationship remained after adjustment for multiple factors including gender, age, history of stroke, history of coronary heart disease, current smoking, current alcohol consumption, physical inactivity, education attainment, CRP, serum uric acid, and BMI. Participants with the HW phenotype were 2.73-fold as likely to have CKD as were those without this phenotype, even after adjust for potential confounders including diabetes and hypertension.

Several potential mechanisms may contribute to the increased CKD risk in subjects with the HW phenotype despite having relatively low BMI level. The HW phenotype is a reliable surrogate marker of visceral obesity. Several studies have indicated that visceral adipose tissue secretes higher quantities of inflammatory cytokines. This was also supported by the finding that, in our study, levels of CRP in the subjects with the HW phenotype are higher than those without this phenotype. Furthermore, visceral obesity or excess fatty acids accompanied with elevated levels of TG may result in accumulation of fat at ectopic tissues including liver, pancreatic b-cells, and the kidney [10, 43]. Ectopic accumulation of fat at liver and pancreatic b-cells may result in steatohepatitis, insulin resistance, and, therefore, hypertension and diabetes [43]. Higher levels of serum uric acid, fasting glucose, higher systolic, and diastolic blood pressure in the participants with the HW phenotype may further support these mechanisms. Besides, ectopic accumulation of fat at kidneys may result in compression of the kidneys and, therefore, unfavourable renal hemodynamic pattern [28]. This, to some degree, may explain the persisted association between the HW phenotype and CKD, even after adjusted for gender, age, BMI, CRP, serum uric acid, diabetes, hypertension, and other potential confounders.

Limitations of our present study should be considered when interpreting these results. First, the sample size of our study was relatively small, especially only 391 men were included. Analyses were thus not carried out separately in men and women, and confidence intervals around the estimates of associations were, therefore, relatively wide. However, we have adjusted gender in the multiple regression analysis. Second, the cross-sectional nature of our study disenabled us to make causal inferences. Third, due to the inconsistent standards in defining the HW phenotype [23, 38], cutoffs of WC and TG that used to define the HW phenotype need to be further validated in different Chinese ethnic groups and across different age.

## Conclusion

The HW phenotype, a validated and convenient marker of visceral obesity, can be a sensitive and useful tool for CKD risk assessment in Chinese, especially those with relatively low BMI. While anthropometric indexes (WC, WHtR, and WHR), surrogate markers of central obesity, failed to identify those subjects with relatively low BMI at high risk of CKD. As indexes on evaluating obesity, the HW phenotype is superior to classical anthropometric obesity indices in discriminating subjects at high risk of CKD and should be used in clinical practice.
